# Variations in Sex Pheromone of the Australian Population of Fall Armyworm: Influence of Age and Mating Status

**DOI:** 10.1007/s10886-025-01607-0

**Published:** 2025-05-17

**Authors:** Sanjana Akter, MD Sahadat Hossain, Rabia Ali, Rajendra Regmi, Soo Jean Park, Bishwo Mainali

**Affiliations:** Applied BioSciences, Macquarie University, Macquarie Park, NSW 2109 Australia

**Keywords:** *Spodoptera frugiperda*, Temporal pattern, Chemical composition, Gland extraction, Headspace volatiles

## Abstract

The rapid establishment of Fall Armyworm (FAW), *Spodoptera* *frugiperda* in Australia necessitates effective and sustainable management strategies. Pheromones offer a promising strategy for mitigating FAW damage through monitoring, mass trapping, and mating disruption. Understanding the pheromone composition of local FAW populations, as well as the variation in pheromone composition and production influenced by the mating status and age of FAW females, provides valuable insights into the factors contributing to pheromone production variability. This study investigated chemical composition of pheromone compounds of FAW population in Australia, temporal pattern of release, and the effect of mating status and age of FAW female on compounds release and production. Pheromone glands were collected by solvent extraction, whereas headspace volatiles were collected by solid phase microextraction (SPME) method. The sample contained four compounds (*Z*)-7-dodecenyl acetate (Z7C12Ac), (*Z*)-9-dodecenyl acetate (Z9C12Ac), (*Z*)-9-tetradecenyl acetate (Z9C14Ac) and (*Z*)-11-hexadecenyl acetate (Z11C16Ac), with Z9C14Ac present in a significantly higher amount. The maximum quantity of compounds from headspace volatiles was released between 4 to 6 h into the scotophase, while the compounds obtained from gland extraction varied across different phases of the scotophase. Younger FAW female released and produced higher amounts of compounds compared to the older individuals. Mated females have significantly higher compounds titre in their pheromone glands compared to the virgins, despite the latter releasing more volatile compounds. These findings highlight the impact of physiological factors on FAW pheromone compounds, offering valuable insights for developing sustainable strategies to manage FAW population in Australia.

**Supplementary Information:**

The online version contains supplementary material available at 10.1007/s10886-025-01607-0.

## Introduction

*Spodoptera frugiperda* (JE. Smith), a Lepidopteran, holometabolous, sporadic moth with a high rate of reproduction commonly known as FAW, originated from tropical and sub-tropical regions of the Americas and is considered a major pest of cereal crops on a global scale (Sparks 1979; Adhikari et al. 2020). FAW is highly polyphagous and is known to cause significant damage to sorghum, sweet corn, maize, rice and various pasture grass from the Poaceae family (Casmuz et al. 2010; Montezano et al. 2018). In 2020, entry of FAW in Australia mainland was confirmed (Maino et al. 2021; Qi et al. 2021) and now is considered as an established pest. The invasion of FAW in Australia occurred through multiple introductions from Southeast Asia (Rane et al. 2023), which may explain the observed genetic variations among populations currently established in the country (Tay et al. 2022a). There are two phenotypically similar strains of this moth that differ in host plant preference and calling behaviour. These are- the corn (C) strain and the rice (R) strain, distinguishable by genetic markers, can interbreed resulting in a hybrid strain (Meagher et al. 2013; Unbehend et al. 2013; Haenniger et al. 2020). It is to be noted that both strains were detected in Australia during the first year of this pest migration, however, the Australian population assessed to date are best characterized as a ‘hybrid strain’ (Rane et al. 2023).

The establishment of FAW in Australia could pose a significant threat to the grain and horticulture industry (Maino et al. 2021), with estimates suggesting that more than 0.8 million ha of cultivable land will be permanently affected within the next ten years, potentially adding an annual operational cost of about $14.2–39.3 million to agricultural producers (Cook et al. 2021). FAW invasion in Australia will potentially lead to an increase in insecticide application to combat the economic damage as insecticide application has remained the main tool to control FAW (Al-Sarar et al. 2006; Blanco et al. 2010). Nonetheless, beyond the reported development of pesticide resistance in FAW (Tay et al. 2022b; Wang et al. 2023), prophylactic and overuse of pesticide exerts significant environment pressure, contributing to biodiversity loss, altered geochemical cycles, freshwater contamination, and climate change impacts (Kaur and Garg 2014).

Australian growers heavily rely on using chemical pesticides to control FAW, however, resistance has already been reported in Australian FAW populations to organophosphate and carbamates (Nguyen et al. 2021), as well as pyrethroids (Bird et al. 2022). In response, a group of research institutions working in a project under the Hort Innovation Australia initiative has identified several parasitoid species capable of attacking FAW eggs and larva (Subramaniam 2022). Additionally, efforts are being made to identify geographical variation of sex pheromone in Australian FAW populations. Despite these efforts, the efficacy of these alternative control strategies has not yet been conclusively validated. Hence, development of a sustainable management strategy is still a demand to mitigate the damage incurred by FAW and to protect agriculture and vulnerable ecosystems. As no single approach can sustainably manage FAW, the use of sex pheromone for early detection, mass trapping and mating disruption is considered a promising strategy to complement other integrated pest management (IPM) tools (Cork 2016; Suckling 2016; McGrath et al. 2018).

Moths are one of the most diverse insect groups and are characterised by an advanced sex pheromone communication system (Cardé and Haynes 2004). The specialized gland of female moth produces and releases a sex pheromone (Löfstedt 1993) that solely attracts males from the same species across a long distance (Cardé and Haynes 2004; Groot et al. 2008). Tumlinson et al. (1986) reported that the sex pheromone of the FAW as a blend of (*Z*)−7-dodecenyl acetate (Z7C12Ac), (*Z*)−9-tetradecenyl acetate (Z9C14Ac), (*Z*)−11-hexadecenyl acetate 11C16Ac), (*Z*)−9-dodecenyl acetate (Z9C12Ac) in the ratio of 0.5: 81: 18: 0.5, correspondingly. Of note, among geographically separated FAW populations, either absence or presence of a specific compound or difference in the relative abundance of the compounds have been documented (Tumlinson et al. 1986; Batista-Pereira et al. 2006; Groot et al. 2008; Lima and McNeil 2009).

Apart from geographically distant populations, the rice and corn strains of FAW also exhibit variations in pheromonal compounds and calling behaviour (Adamczyk et al. 1997; Lima and McNeil 2009; Unbehend et al. 2013). Furthermore, pheromone production and release in moths is influenced by scotophase, age, mating status, and sex (Jaffe et al. 2007; Harari et al. 2011; Li et al. 2012; Wang et al. 2015; De Pasqual et al. 2024). For example, nocturnal females exhibit higher pheromone titres during scotophase, likely supporting reproductive activities, with titres peaking early and declining over time (Raina et al. 1986; Lu et al. 2017). Mated and younger females generally produce more pheromones than virgins and older females, reflecting changes in fertility and receptivity (Torres-Vila et al. 2002; Levi-Zada et al. 2019). Pheromone composition can also vary depending on the process of pheromone extraction. In FAW populations collected from Florida, two aldehydes were present in the pheromone glands, but were absent in the volatiles collected from calling females (Tumlinson et al. 1986). However, one of these two aldehydes suppress the FAW male capturing rate, suggesting that it is not a pheromone compound despite their presence in the glands.

As aforementioned, variations in pheromonal components among Australian FAW populations are likely, and could influence pest behaviour, host preferences, and sexual communication. These differences highlight the need to optimize lure formulations for improved FAW monitoring and/or mass trapping systems. However, to the best of our knowledge, this is the first study in Australia to identify the pheromone compositions of Australian FAW populations or to investigate the rhythmicity of pheromone production and release in relation to age and mating status. Such insights would provide a scientific foundation for effective pest management designed for Australian FAW populations and environmental conditions.

This study characterised the volatiles emitted by calling female and the pheromone gland extract of a FAW population in Australia, determined the temporal pattern of pheromone production and release, and investigated the influence of age and mating status on pheromone production and release in FAW females.

## Methods and Materials

### Moth Rearing

FAW population used in this study were obtained from a colony initially established at Commonwealth Scientific and Industrial Research Organisation (CSIRO), originally collected from maize field belongs to the Northern Australian Crop Research Alliance Pty Ltd in Kununurra, Western Australia (15.6953°S, 128.7335°E; elevation 36.1 m). It has been maintained in a controlled environment room (CER) in the Entomology Laboratory, Applied BioSciences, Macquarie University, Australia since 2022. The set temperature in the CER is 25 ± 1 °C, relative humidity is 65 ± 5%, light intensity is 0.4 lum/ft^2^ at dark phase, whereas 90 ± 2 lum/ft^2^ at light phase and at an inverted photocycle period is light: dusk: dark: dawn (13.5: 0.5: 9.5: 0.5 h). Each larva was reared individually in the Petri dishes containing a semi-solid artificial diet, which was prepared following the procedure of Apirajkamol et al. (2020). Pupae were sexed by following the identification keys mentioned by Block et al. (2021). Male and female pupae were maintained in separate rearing cages, with each cage housing adult moths of the same sex and age. Adults were supplied with a 10% (w/v) honey solution as a food source.

### Headspace Volatiles Collection

The collection of headspace volatiles of FAW females was performed in a CER with the same photocycle, temperature and humidity as the rearing room. A single female was placed in each headspace vial (20 mL) at the start of the dusk period, and the vial was sealed with a steel screw cap (Shimadzu Corporation, Nakagyo-ku, Kyoto, Japan) to prevent volatile loss. The vial was kept in the CER to allow females to release volatiles within it. After the designated volatiles collection period in each experiment described below, the vials, containing the females, were placed at −30 ºC and stored until analysed by Gas chromatography–mass spectrometry (GC–MS).

### Temporal Patterns of Volatile Release in the Headspace of Virgin Females

To study the temporal pattern of volatiles release from calling virgin females, headspace volatiles were collected every two hours from the beginning of the scotophase and continuing until the 8th hour. This collection interval was based on previous studies on pheromone release patterns, which indicated that females start releasing pheromones 2–4 h into the scotophase (Tumlinson et al. 1986). The headspace volatiles of 20 females were collected for each 2-h period.

### Effect of Mating on the Volatiles Released by Calling Females

To study the influence of mating status on the volatiles released by calling FAW females, headspace volatiles were collected from virgin and mated females (*n* = 15 each) over a 5-h period, starting from the onset of the scotophase. Mated females were obtained by pairing a two-day old virgin female with a two-day old virgin male in a plastic cage and observing them throughout the scotophase to confirm copulation. Once copulation finished, the mated females were separated to prevent remating and kept in a separate cage for headspace volatile collection.

### Effect of Age on the Volatiles Released by Calling Females

To study the effect of age on the sex volatiles released by calling females, headspace volatiles were collected from one-, four- and seven-day old females over a 5-h period, starting at the onset of dusk period. Volatiles were collected from 15 females in each age group.

### Analysis of Headspace Volatiles by Automated SPME–GC–MS

An internal standard, 1-octanol, was used to compare the amount of pheromone components quantitatively. The stock solution of internal standard (0.20 mg/mL) was prepared by dissolving 1-octanol (19.99 mg) in reverse osmosis water in a volumetric flask (100 mL), which was diluted to give a 0.04 mg/mL solution for sample analysis. An aqueous solution of 1-octanol was chosen as the internal standard, as negligible affinity of SPME fibres to water has been utilized in a range of SPME analysis (Stiles et al. 2008). The solubility of 1-octanol, 0.49 mg/mL (Šegatin and Klofutar 2004) adequately covers the analytical concentration range. To validate the reliability of the internal standard in the employed method, we analysed an additional 18 female headspace samples for reproducibility, intraday and interday variations. These samples were collected using the same headspace volatile collection method described above. The results of validation parameters are presented in Table S1 in Supplementary Information.

All samples were analysed using Shimadzu GC-2030 gas chromatograph, Shimadzu GCMS-QP2020 NX mass spectrometer with a single quadrupole mass filter, and a Shimadzu AOC-6000 autosampler (Shimadzu Corporation, Nakagyo-ku, Kyoto, Japan). The instrument was equipped with a splitless/split injector, a Shimadzu SH-Rtx-5Sil MS fused silica capillary column (30 m × 0.25 mm, 0.25 μm film). Helium (BOC, North Ryde, NSW, Australia) (99.999%; Grade 5.0) was used as the carrier gas with a constant flow rate of 1.49 mL/min.

For adsorption of pheromone compounds and the internal standard, Shimadzu SPME Arrow (DVB/PDMS, sorption phase thickness 120 µm) (Shimadzu Corporation, Nakagyo-ku, Kyoto, Japan) was used. The autosampler parameters were as follows: the fiber was conditioned at 260 ºC for 5 min; the sample vial was incubated at 60 ºC for 5 min; the agitator speed was 250 rpm; the stirrer speed was 1000 rpm; the sample adsorption time was 5 min; the internal standard adsorption time was 1.8 sec; and the sample desorption time was 1.5 min.

The samples were analysed in split mode with a 3:1 ratio. The initial column temperature was set at 60 ºC and held for 2 min, then increased to 255 ºC at a rate of 15 ºC/min and held for 1 min. The injector and MS transfer line temperatures were both set at 250 ºC. The ionisation method was electron impact at 70 eV, with an ion source temperature of 200 ºC. The MS was operated in Fast Automated Scan/SIM Type mode. Based on the mass fragmentation patterns of each compound, SIM fragments were chosen with the following target and qualifier ions: (Z7C12Ac: target ion m/z 166, qualifier ion m/z 82 and 68; Z9C12Ac: target ion m/z 166, qualifier ion m/z 81 and 67; Z9C14Ac: target ion m/z 194, qualifier ion m/z 96 and 82; Z11C16Ac: target ion m/z 222, qualifier ion m/z 96 and 82). The dwell time for each ion was set to 200 ms, providing sufficient sensitivity while maintaining an adequate number of data points across chromatographic peaks. GCMS Postrun Analysis software version 4.45 was used to process the data and obtain the peak areas of the target compounds and internal standard. The GC response of the internal standard (1-octanol) remained within analytically acceptable limits throughout the experiment. To compare absolute compound levels between groups, GC responses were normalized by dividing the peak area of each target compound by the peak area of the internal standard and used for statistical analysis.

### Pheromone Gland Extraction

Based on the results from the previous section on the temporal patterns of volatiles release it was determined that the peak release occurs between the 4th and 6th hour of the scotophase. To assess the effect of mating status and age on gland extracts during this period, the pheromone glands of females were excised between the 4th and 6th hour of the scotophase. Under a stereoscope, the pheromone gland was extracted from the live female moth by gently squeezing the tip of the abdomen and pulling the pheromone gland out with fine forceps. The extracted pheromone glands were collected in a 250 μL glass insert placed into a 2 mL glass vial that was kept on dry ice. Once five pheromone glands were collected, the vial was removed from dry ice, and 100 μL of *n-*hexane was added to the glands in the vial. The vial was kept standing for approximately 15 min at room temperature (25 ºC). After 15 min, using a micropipette, the extract was transferred to another 250 μL glass insert in a standard GC vial. Then the samples were stored at −30 ºC until analysed.

### Temporal Pattern of Pheromone Gland Compound Production in Virgin Females

To study the temporal pattern of pheromone gland compounds production in virgin females, glands were excised every two hours from the start of the scotophase and continuing until the 8th hour of the scotophase. The pheromone glands from five different females were pooled together as one replication, with a total of 10 replications.

### Effect of Mating Status on the Pheromone Gland Contents

To determine the effect of female mating status on the pheromone gland content, the pheromone gland from three-day old virgin and mated females was removed and extracted, where five pheromone glands represent a single replication, with a total of 10 replications. Mated females were obtained by the same method described in the section of effect of mating on the volatiles released by calling females.

### Effect of Age on the Pheromone Gland Contents

To investigate the effect of female age on the pheromone gland content, the pheromone glands of one-, four- and seven-day old virgin females were excised and extracted. Ten replications were collected for each studied group where five pheromone glands represent a single replication.

### Analysis of Pheromone Gland Extracts by GC–MS

(i) *GC–MS Conditions.* The general GC–MS conditions with EI as ionisation were the same as those used in the headspace analysis above, except the temperature program: the initial column temperature was set at 80 ºC, then increased to 240 ºC at a rate of 10 ºC/min, held for 2 min, and further increased to 300 ºC at a rate of 30 ºC/min. For the qualitative confirmation of the pheromone gland components, the natural gland extract was also analysed by GC–MS with chemical ionisation (CI). The samples were analysed in positive ion mode with methane gas (ultrapure, BOC, Australia) as the reagent gas. All other GC–MS conditions were the same as those used in the analysis with EI, except that spectra were obtained over a mass range of *m/z* 200–400.
(ii) *Preparation of Standard Solutions.* Stock solutions for the four pheromone components, namely (*Z*)−7-dodecenyl acetate (Z7C12Ac), (*Z*)−9-dodecenyl acetate (Z9C12Ac), (*Z*)−9-tetradecenyl acetate (Z9C14Ac), and (*Z*)−11-hexadecenyl acetate (Z11C16Ac), were prepared by dissolving 17.5 mg, 18.9 mg, 22.0 mg, and 22.3 mg, respectively, in n-hexane in 10 mL volumetric flasks. The stock solutions were then diluted to give the following concentrations: Z7C12Ac (1.8 μg/mL), Z9C12Ac (1.9 μg/mL), Z9C14Ac (22.0 μg/mL), Z11C16Ac (4.4 μg/mL). These secondary stock solutions were further diluted to give the concentration ranges comparable to their proportions found in natural gland extracts; Z7C12Ac (0.03–1.8 μg/mL), Z9C12Ac (0.03–1.9 μg/mL), Z9C14Ac (0.17–22.0 μg/mL), Z11C16Ac (0.07–4.4 μg/mL). A stock solution of n-heptadecane (5.06 μg/mL) was prepared to serve as the internal standard. An aliquot of 5 μL of the internal standard was incorporated into the standard solutions and samples, resulting in a final concentration of 0.24 μg/mL throughout the experiment.
(iii) *Quantification of the Pheromone Compounds *Via* Standard Curves.* To estimate the concentration of compounds present in the sample solutions, the standard solutions were analysed by GC–MS, generating a standard curve for each compound. The concentration of each compound was then determined based on these standard curves. Subsequently, the amount of each compound in one moth was estimated by taking account of the original volume (105 µL) and the number of moths per sample (*n* = 5).

#### Data Analysis

For the statistical analyses and graphical presentations, the R software (version 4.4.2) was used. The normality of compound data was assessed using Shapiro’s test. If the data deviated from normality, the *bestNormalize* package in R was used to apply the *orderNorm* transformation for normalization. An analysis of variance (*ANOVA*) was conducted to compare the temporal pattern of volatiles released by calling female and pheromone gland content within the scotophase, followed by *Tukey’s* test post hoc analysis for multiple mean comparisons. To compare the difference of headspace volatiles and pheromone gland content among females of three different age groups, an *ANOVA* followed by *Tukey’s* test post hoc analysis for multiple mean comparisons was conducted. A *Welch’s t*-*test* was conducted to compare quantitatively the headspace volatiles and pheromone gland content and the individual compound between virgin and mated females. To compare the relative amount of compounds released and content by FAW female among the variables an *ANOVA* was conducted followed by *Tukey’s test* post hoc analysis.

## Results

### Characterisation of Pheromone Compounds of FAW Females

**Fig. 1:**
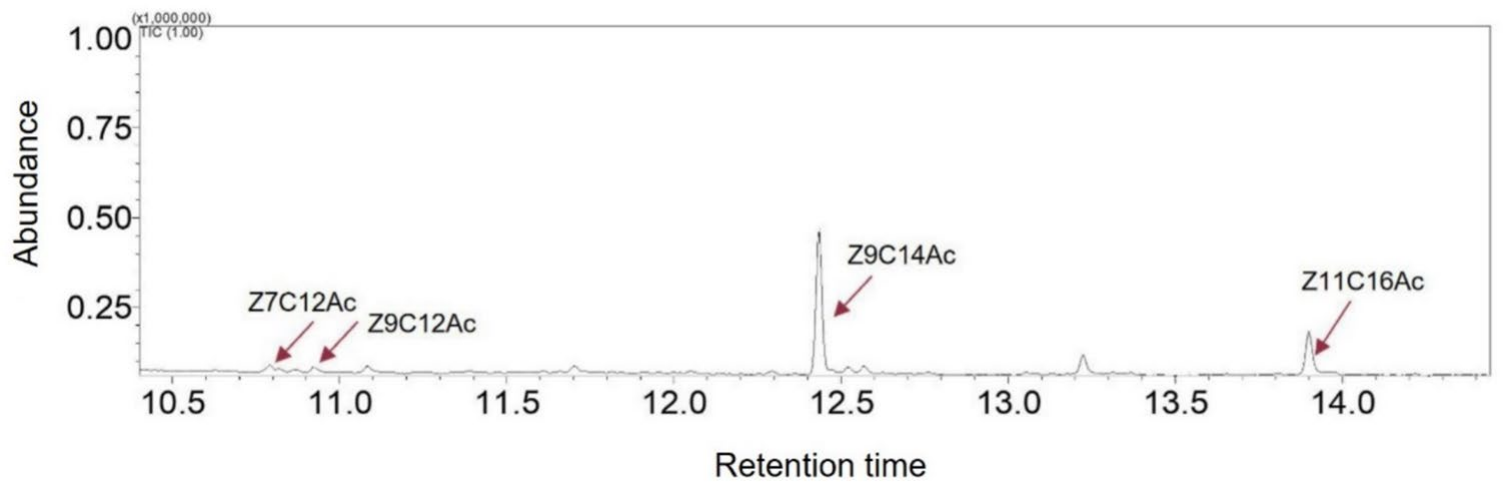
GC–MS chromatogram profile of volatiles collected from calling FAW female. (*Z*)−7-dodecenyl acetate (Z7C12Ac), (*Z*)−9-dodecenyl acetate (Z9C12Ac), (*Z*)−9-tetradecenyl acetate (Z9C14Ac) and (*Z*)−11-hexadecenyl acetate (Z11C16Ac)

The sex pheromone of female moths from the colony initially collected from Western Australia was found to contain four compounds: (*Z*)−7-dodecenyl acetate (Z7C12Ac), (*Z*)−9-dodecenyl acetate (Z9C12Ac), (*Z*)−9-tetradecenyl acetate (Z9C14Ac) and (*Z*)−11-hexadecenyl acetate (Z11C16Ac). The compounds were identified by comparing their retention times on the GC column with those of synthetic standards. The major compound was Z9C14Ac, whereas the other three compounds were present in lower quantities (Fig. 1). Further confirmation by GC–MS analysis using chemical ionisation (CI) confirmed *MH* + ions (protonated molecular ion) for each compound, while molecular ions were typically absent in the mass spectra obtained through electron impact ionisation (EI) (Supplementary Fig. 1).

### Temporal Patterns of Volatile Release in the Headspace of Virgin Females

**Fig. 2:**
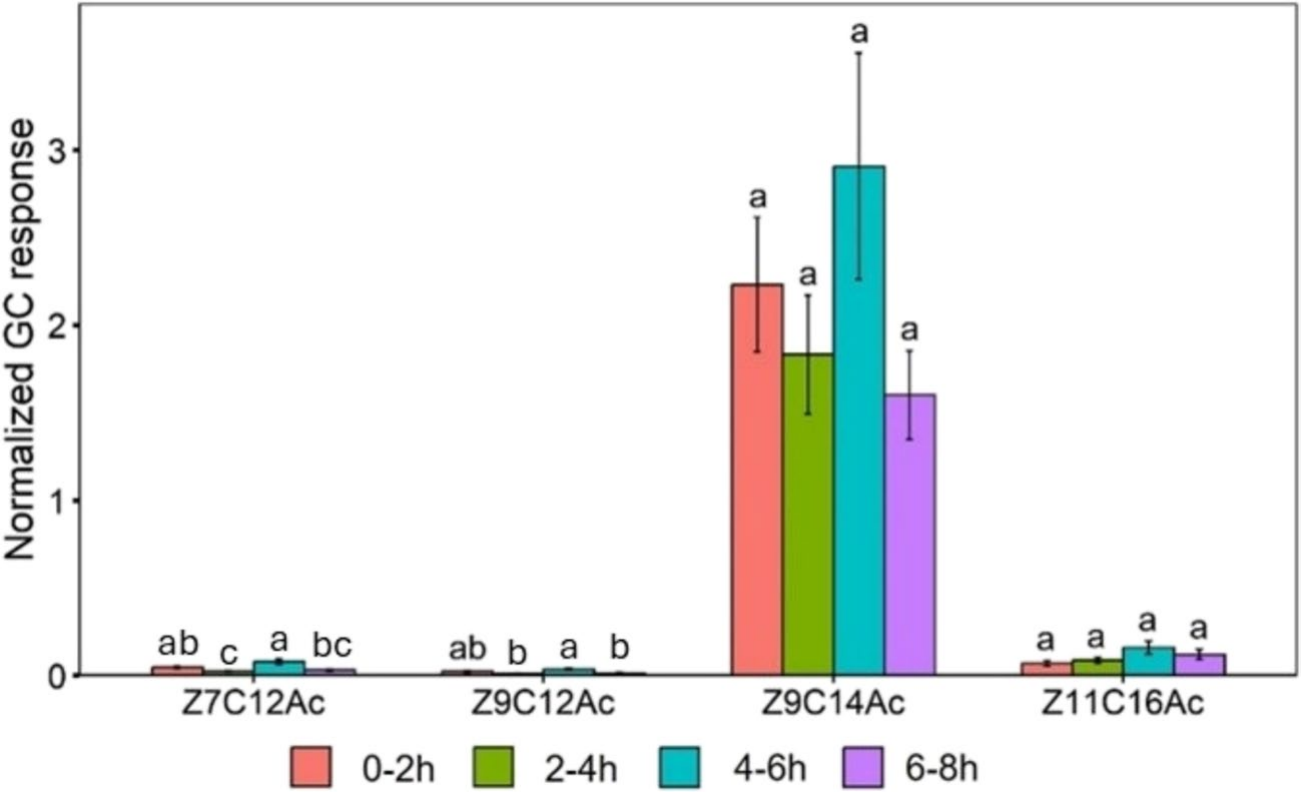
Amount of headspace volatile (Mean ± SE) released by FAW virgin females at different time of scotophase. *ANOVA* conducted followed by *Tukey’s* test post hoc analysis at *P* < 0.05 level of significance. The different lowercase letters indicate significance at *P* < 0.05; same lowercase letters indicate non-significant (*P* ≥ 0.05) for each compound (*n* = 20 for each group of females)

The volatile compounds Z7C12Ac (F_3,76_ = 9.174, *P* < 0.001) and Z9C12Ac (F_3,76_ = 6.098, *P* < 0.001) released by virgin female varied significantly across different time points within the scotophase. However, there was no significant effect of time on the release of Z9C14Ac (F_3,76_ = 0.925, *P* > 0.05) and Z11C16Ac (F_3,76_ = 1.26, *P* > 0.05). Figure 2 illustrates these variations along with the results of *Tukey’s* post hoc analysis. *Tukey’s* test confirmed Z7C12Ac and Z9C12Ac release was significantly higher between the 4th-6th hour compared with 2nd-4th hour of scotophase (*P* < 0.001). The highest release of the compounds was between the 4 th-6 th of the scotophase while the lowest release was between 2nd-4th of the scotophase.

The relative proportion of Z7C12Ac release was varied significantly across different time points within the scotophase (F_3,76_ = 3.845, *P* < 0.05). Table 1 represents these variations along with the results of *Tukey’s* post hoc analysis. *Tukey’s* test confirmed Z7C12Ac release was significantly higher between the 0-2nd hour compared with 2nd-4th hour of scotophase (*P* < 0.05). However, there was no significant effect of time on the relative proportion of Z9C12Ac (F_3,76_ = 0.785, *P* > 0.05), Z9C14Ac (F_3,76_ = 0.368, *P* > 0.05) and Z11C16Ac (F_3,76_ = 0.157, *P* > 0.05).

**Table 1:** Relative proportion of headspace volatiles and pheromone gland extracts of FAW female

Variables	Treatments	Relative proportion of volatile compounds released (%)			
		Z7C12Ac	Z9C12Ac	Z9C14Ac	Z11C16Ac
Temporal pattern	0–2 h	4.12^a^	4.07^a^	85.80^a^	6.01^a^
	2–4 h	1.36^b^	5.28^a^	88.49^a^	4.86^a^
	4–6 h	2.95^ab^	6.40^a^	85.69^a^	4.96^a^
	6–8 h	2.80^ab^	3.41^a^	88.03^a^	5.75^a^
		*P* < 0.05	*P* > 0.05	*P* > 0.05	*P* > 0.05
Mating status	Virgin	1.97^b^	4.58^b^	89.48^a^	3.97^b^
	Mated	11.97^a^	25.23^a^	53.28^b^	9.52^a^
		*P* < 0.05	*P* < 0.01	*P* < 0.01	*P* < 0.05
Age	One-day	3.25^a^	10.52^a^	82.82^a^	3.42^b^
	Four-day	3.43^a^	18.60^a^	73.54^a^	4.43^b^
	Seven-day	1.66^a^	12.11^a^	78.12^a^	8.12^a^
		*P* > 0.05	*P* > 0.05	*P* > 0.05	*P* < 0.01
Relative proportion of gland extract (%)					
Temporal pattern	0–2 h	3.54^a^	2.87^a^	80.55^a^	13.04^b^
	2–4 h	2.57^b^	1.83^b^	78.02^ab^	17.58^b^
	4–6 h	2.70^b^	2.00^b^	78.62^ab^	16.67^b^
	6–8 h	2.72^b^	1.79^b^	76.63^b^	18.87^a^
		*P* < 0.001	*P* < 0.001	*P* < 0.01	*P* < 0.001
Mating status	Virgin	2.68^a^	1.94^a^	78.73^a^	16.65^a^
	Mated	2.03^b^	1.06^b^	81.44^a^	15.48^a^
		*P* < 0.01	*P* < 0.01	*P* > 0.05	*P* > 0.05
Age	One-day	2.51^a^	1.38^b^	79.66^a^	16.45^b^
	Four-day	3.03^a^	2.60^a^	74.59^a^	19.78^ab^
	Seven-day	3.09^a^	2.34^a^	70.13^b^	24.43^a^
		*P* > 0.05	*P* < 0.01	*P* < 0.001	*P* < 0.01

*ANOVA* conducted followed by *Tukey’s* test post hoc analysis at *P* < 0.05 level of significance. Different lowercase letter showed significant differences, whereas same letter denotes non-significant

### Effect of Mating on the Volatiles Released by Calling Females

**Fig. 3:**
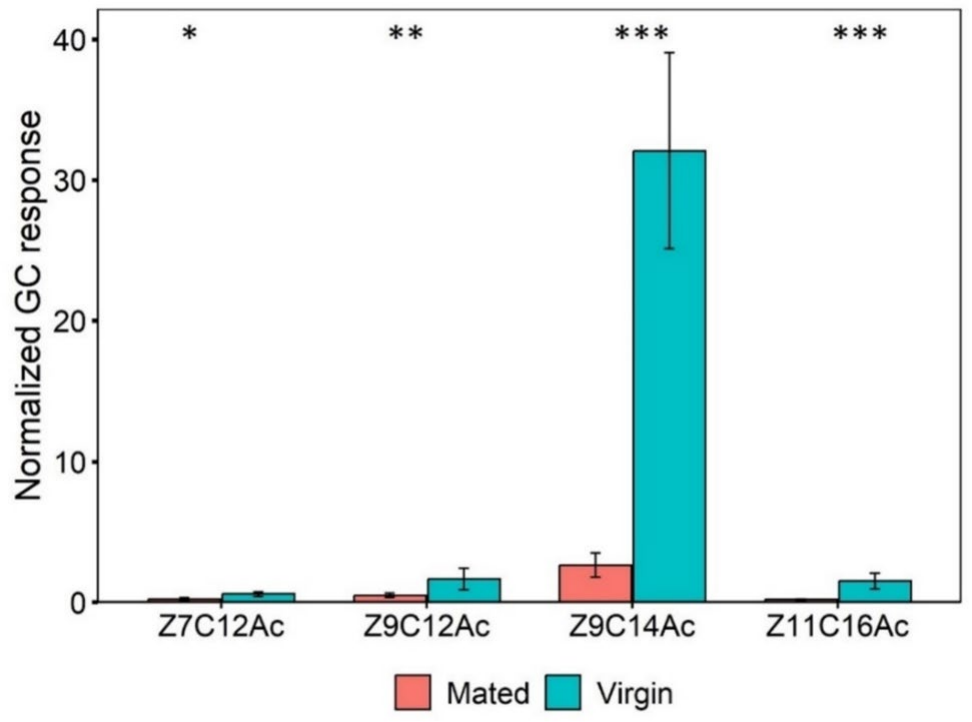
Amounts of headspace volatiles (Mean ± SE) released by mated and virgin FAW females. A *Welch’s t-test* was conducted to compare quantitatively. Asterisks indicate significance for each pheromone compound. (^***^P < 0.001; ^**^P < 0.01; ^*^P < 0.05) (*n* = 15 for each group of females)

The release of volatile compounds varied significantly between virgin and mated females: Z7C12Ac (t = −2.5413, df = 23.995, *P* < 0.05), Z9C12Ac; (t = −2.9687, df = 22.881, *P* < 0.01), Z9C14Ac (t = −6.7312, df = 24, *P* < 0.001) and Z11C16Ac (t = −4.9955, df = 23.995, *P* < 0.001).The release of all compounds were significantly higher in virgin females compared to mated females (Fig. 3).

Significantly higher proportion of Z7C12Ac was released in mated females (t = 2.3972, df = 12.207, *P* < 0.05), Z9C12Ac (t = 3.0529, df = 12.76, *P* < 0.01) and Z11C16Ac (t = 2.8368, df = 18.286, *P* < 0.05) compared to virgin female. Whereas Z9C14Ac (t = −4.1478, df = 12.6, *P* < 0.01) was released in significantly higher proportion in virgin female than mated one (Table 1).

### Effect of Age on the Volatiles Released by Calling Females

**Fig. 4:**
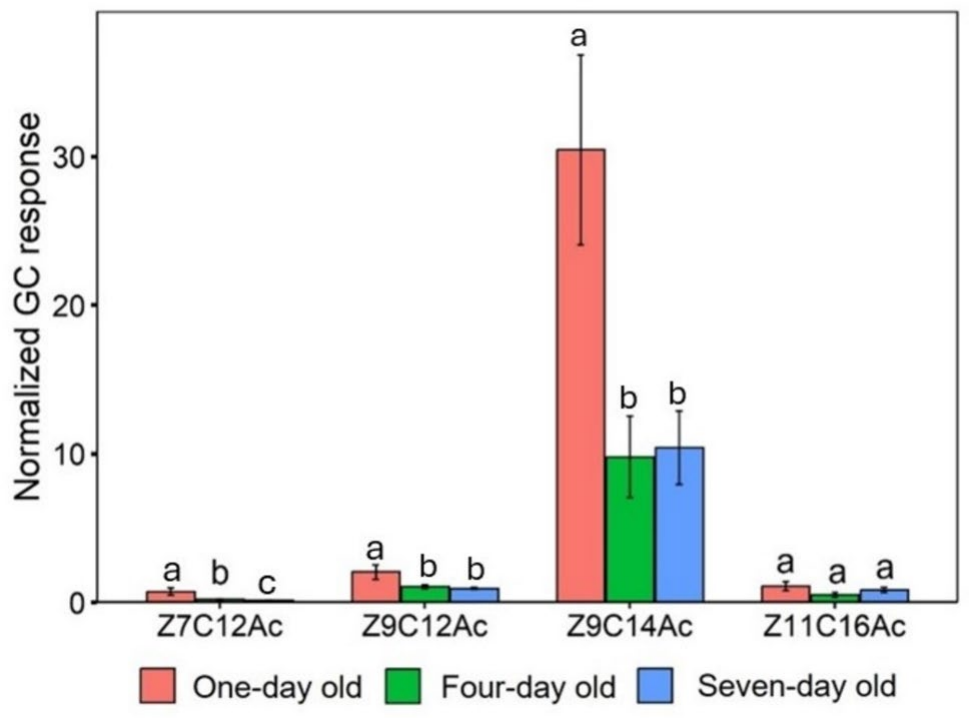
Amount of headspace volatiles (Mean ± SE) released by FAW females of different ages. *ANOVA* conducted followed by *Tukey’s* test post hoc analysis at *P* < 0.05 level of significance. The different lowercase letters indicate significance at *P* < 0.05; same lowercase letters indicate non-significant (*P* ≥ 0.05) for each pheromone compound (*n* = 15 for each group of females)

The release of volatile compounds varied significantly among all three age groups. Z7C12Ac (F_2,42_ = 25.31, *P* < 0.001), Z9C14Ac (F_2,42_ = 6.21, *P* < 0.01) and Z9C12Ac (F_2,42_ = 8.187, *P* < 0.001). However, age has no significant effect on the release of Z11C16Ac (F_2,42_ = 1.916, *P* > 0.05). Figure 4 illustrates these variations along with the results of *Tukey’s* post hoc analysis. *Tukey’s* test confirmed Z7C12Ac release was significantly higher in one-day old female headspace compared with four-day and seven-day old headspace and four-day old was higher compared with seven-day old female headspace (*P* < 0.001). Z9C12Ac and Z9C14Ac release were significantly higher in one-day old female headspace compared with four-day and seven-day old female headspace (*P* < 0.001 and *P* < 0.01 respectively). The highest release of compounds was in one-day old female headspaces.

The relative proportion of Z11C16Ac varied significantly among all three age groups (F_2,42_ = 5.67, *P* < 0.01). However, age has no significant effect on the relative proportion of Z7C12Ac (F_2,42_ = 1.77, *P* > 0.05), Z9C14Ac (F_2,42_ = 1.1, *P* > 0.05) and Z9C12Ac (F_2,42_ = 1.58, *P* > 0.05). Table 1 represents these variations along with the results of *Tukey’s* post hoc analysis. *Tukey’s* test confirmed seven-day old female headspace released significantly higher proportion of Z11C16Ac compared with one -day and four-day old headspace (*P* < 0.01).

### Temporal Pattern of Pheromone Gland Compound Production in Virgin Females

**Fig. 5:**
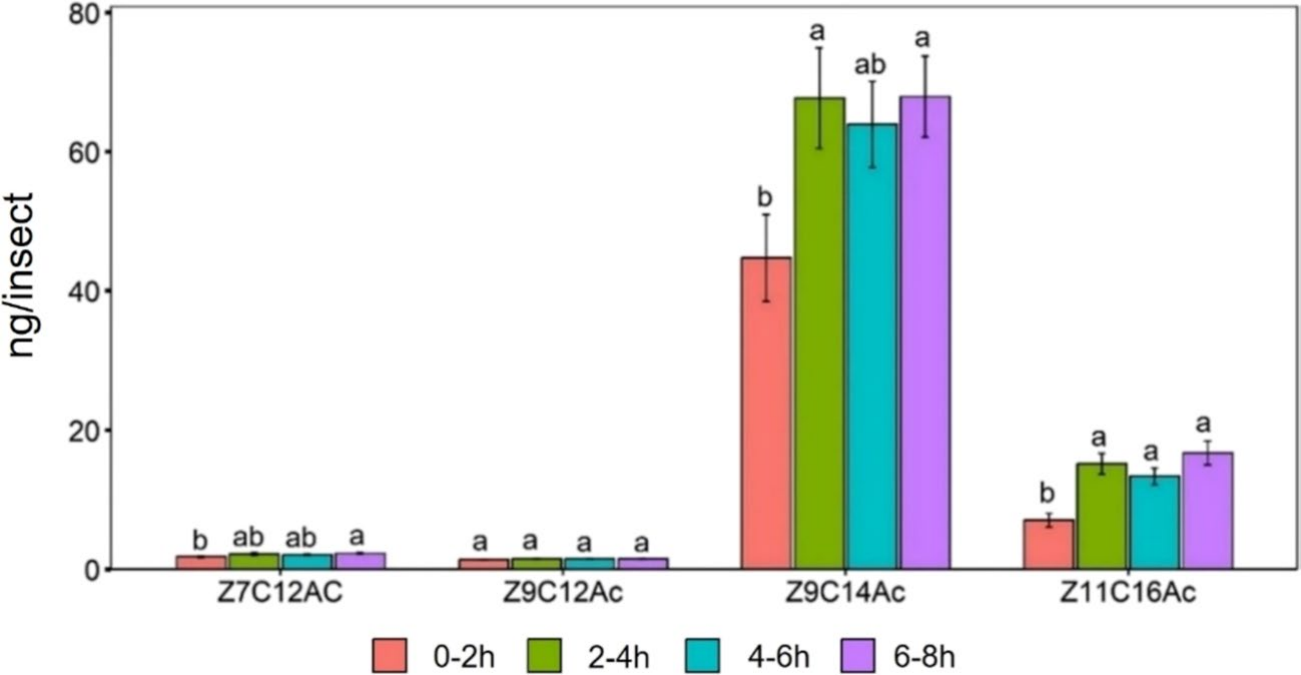
Amount of pheromone gland compounds (Mean ± SE) extracted from FAW females at different times of the scotophase. *ANOVA* conducted followed by *Tukey’s* test post hoc analysis at *P* < 0.05 level of significance. The different lowercase letters indicate significance at *P* < 0.05; same lowercase letters indicate non-significant (*P* ≥ 0.05) for each pheromone compound (*n* = 10, the number of replications)

The production of compounds in the pheromone gland of virgin females varied significantly across different time points within the scotophase: Z7C12Ac (F_3,36_ = 4.097, *P* < 0.05), Z9C14Ac (F_3,36_ = 3.967, *P* < 0.05) and Z11C16Ac (F_3,36_ = 12.53, *P* < 0.001). However, there was no significant effect of time on the amount of Z9C12Ac (F_3,36_ = 0.955, *P* > 0.05). Figure 5 illustrates these variations along with the results of *Tukey’s* post hoc analysis. *Tukey’s* test confirmed Z7C12Ac production was significantly higher between the 6th-8th hour compared with 0-2nd hour of scotophase (*P* < 0.05). Z9C14Ac production was significantly higher between the 2nd-4th and the 6th-8th hours compared with the 0-2nd hour of scotophase (*P* < 0.05). Z11C16Ac production was significantly higher at all three time points compared to the 0-2nd hour (*P* < 0.05). The highest production of all the compounds were between the 6th-8th hour of the scotophase while the lowest production was between 0-2nd hour of the scotophase.

The relative proportion of compounds in the pheromone glands of FAW females varied significantly across different time points within the scotophase: Z7C12Ac (F_3,36_ = 8.143, *P* < 0.001), Z9C12Ac (F_3,36_ = 7.762, *P* < 0.001), Z9C14Ac (F_3,36_ = 4.481, *P* < 0.01) and Z11C16Ac (F_3,36_ = 13.51, *P* < 0.001). Table 1 represents these variations along with the results of *Tukey’s* post hoc analysis. *Tukey’s* test confirmed the relative proportion Z7C12Ac, Z9C12Ac and Z11C16Ac was significantly higher between the 0-2nd hour compared with 2nd-4th, 4th-6th and 6th-8th hour of scotophase (*P* < 0.001). The relative proportion Z9C14Ac was significantly higher between the 0-2nd compared with the 2nd-4th and 4th-6th and significantly lower between 6th-8th hour of scotophase (*P* < 0.01).

### Effect of Mating Status on the Pheromone Gland Contents

**Fig. 6:**
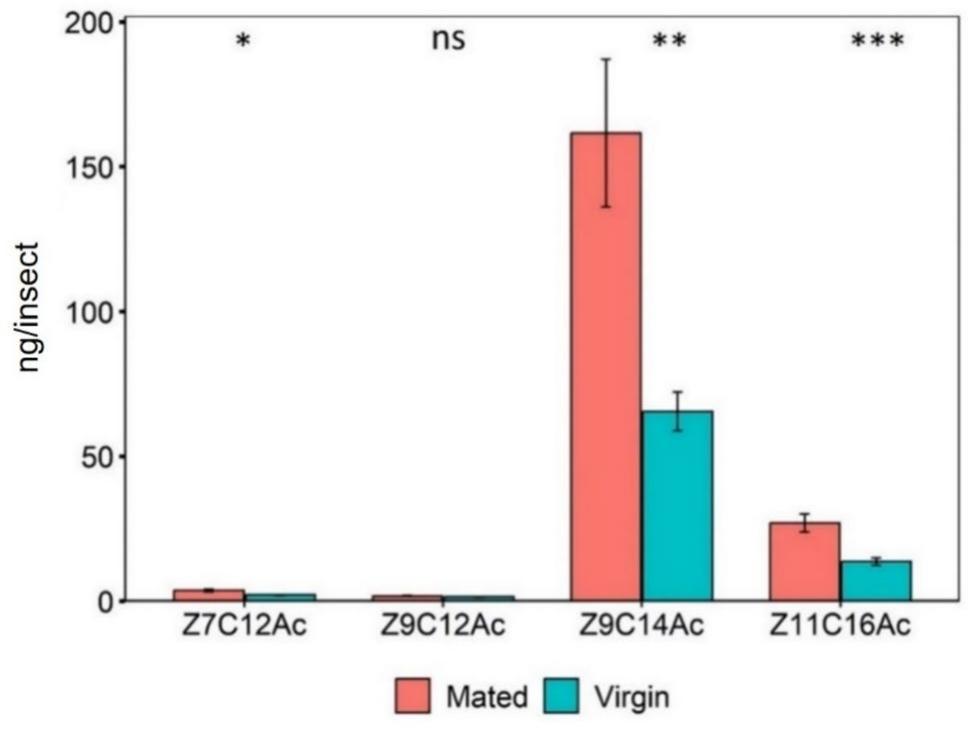
Amount of pheromone gland compounds (Mean ± SE) contained in mated and virgin FAW females. A *Welch’s t-test *was conducted to compare quantitatively. The asterisks indicate significance (^***^P < 0.001; ^**^P < 0.01; ^*^P < 0.05; ^ns^non-significant *P* ≥ 0.05) for each pheromone compound (*n* = 10, the number of replications)

Mated females had significantly higher quantities of each compound in the pheromone glands than in virgin females, except for the compound Z9C12Ac, as confirmed by a welch’s t-test (Fig. 6). Mated females contained significantly higher quantities of the major compound, Z9C14Ac (mated:161.58 ng/gland, virgin: 61.87 ng/gland; t = 3.2114, df = 15.198, *P* < 0.01), and the minor compounds Z7C12Ac (mated: 3.75 ng/gland, virgin: 2.07 ng/gland; t = 2.4325, df = 11.535, *P* < 0.05) and Z11C16Ac (mated: 26.99 ng/gland, virgin: 12.74 ng/gland; t = 4.8151, df = 15.878, *P* < 0.001).There were no significant differences in the amount of Z9C12Ac in the pheromone gland when comparing females (mated: 1.76 ng/gland, virgin: 1.54 ng/gland) (t = 1.9708, df = 15.392, *P* > 0.05).

Significantly higher proportion of Z7C12Ac was found in virgin females (t = −3.0267, df = 14.039, *P* < 0.01) and Z9C12Ac (t = −3.9362, df = 15.605, *P* < 0.01) compared to mated females. However, there were no significant differences in the proportion of Z9C14Ac (t = 1.1925, df = 9.5557, *P* > 0.05) and Z11C16Ac (t = −0.55876, df = 9.2652, *P* > 0.05) in both female (Table 1).

### Effect of Age on the Pheromone Gland Contents

**Fig. 7:**
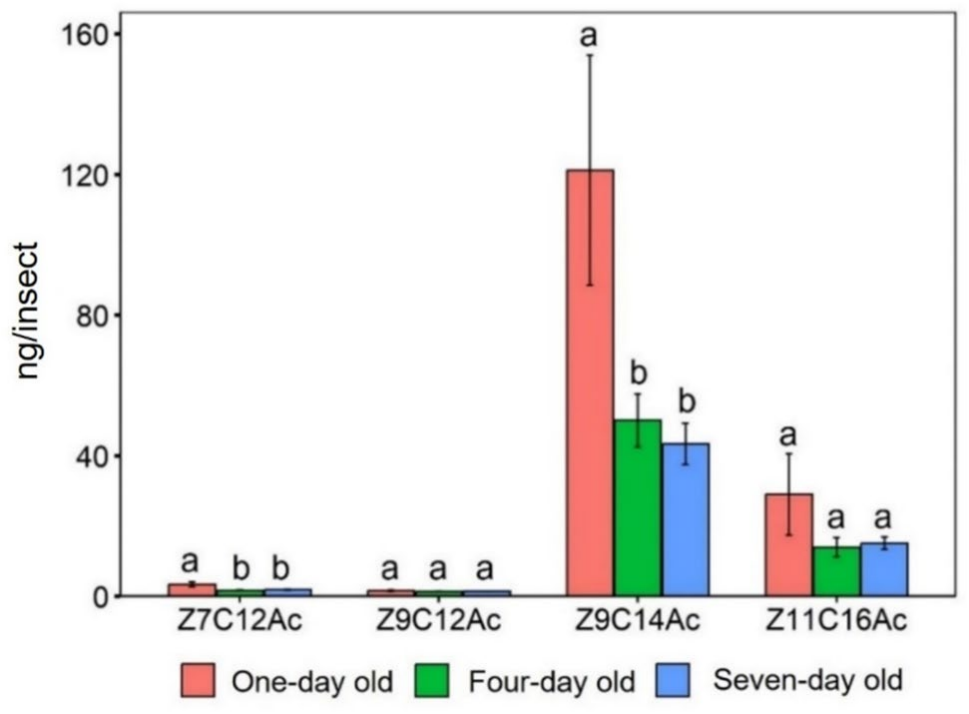
Amount of pheromone gland compounds (Mean ± SE) contained in virgin FAW females of different ages. *ANOVA* conducted followed by *Tukey’s* test post hoc analysis at *P* < 0.05 level of significance. The different lowercase letters indicate significance at *P* < 0.05; same lowercase letters indicate non-significant (*P* ≥ 0.05) for each pheromone compound (*n* = 10, the number of replications)

The production of compounds in the pheromone glands of female FAW varied significantly among three different age groups: Z7C12Ac (F_2,27_ = 15.75, *P* < 0.001), Z9C14Ac (F_2,27_ = 9.901, *P* < 0.001). However, age has no significant effect on the amount of Z9C12Ac (F_2,27_ = 2.361, *P* > 0.05) and Z11C16Ac (F_2,27_ = 2.055, *P* > 0.05). Figure 7 illustrates these variations along with the results of *Tukey’s* post hoc analysis. *Tukey’s test* confirmed Z7C12Ac and Z9C14Ac production was significantly higher in the gland of in one-day old female compared to four-day and seven-day old (*P* < 0.001).

The relative proportion of compounds in the pheromone glands of female varied significantly among three different age groups: Z9C14Ac (F_2,27_ = 11.5, *P* < 0.001), Z9C12Ac (F_2,27_ = 5.64, *P* < 0.01) and Z11C16Ac (F_2,27_ = 7.398, *P* < 0.01). However, age has no significant effect on the amount of Z7C12Ac (F_2,27_ = 2.403, *P* > 0.05). Table 1 represents these variations along with the results of *Tukey’*s post hoc analysis.

## Discussion

This study, for the first time, reports on the four candidate pheromone compounds identified in the headspace volatiles and pheromone glands of Western Australian (WA) FAW population and additionally demonstrates that mating status and age significantly affect the release and production of the identified compounds in female FAW. Female moth sex pheromones, detected by males, serve as a well-studied model for understanding sexual attraction through pheromone mediation (Karlson and Butenandt 1959; Tumlinson et al. 1986; Zhang et al. 2015). Several studies have documented intra-specific variation in moth sex pheromones, influenced by physiological and environmental factors, leading to differences in both titre and composition (Raina et al. 1991; Li et al. 2015; Wang et al. 2015).

We confirmed that the volatiles and pheromone gland extract of female FAW from the WA population contained four compounds, including (*Z*)−7-dodecenyl acetate (Z7C12Ac), (*Z*)−9-dodecenyl acetate (Z9C12Ac), (*Z*)−9-tetradecenyl acetate (Z9C14Ac), and (*Z*)−11-hexadecenyl acetate (Z11C16Ac) with Z9C14Ac being the most abundant. These findings are consistent with previous studies on FAW sex pheromones from different geographic locations. The four compounds found in our study align with the findings of Tabata et al. (2023) and Unbehend et al. (2013), where Z9C14Ac, Z11C16Ac, Z7C12Ac and Z9C12Ac were identified from the pheromone gland of FAW female in Japan and Florida respectively, although the proportions of the compounds are different (Table 1).

There is evidence suggesting that the number and the variation in the proportion of the compounds in different FAW populations could potentially indicate geographic (Tumlinson et al. 1986; Cruz-Esteban et al. 2018) and strain specific differences (Groot et al. 2008; Lima and McNeil 2009). Groot et al. (2008) reported that the pheromone gland of the rice strain FAW females exhibited a significantly lesser amount of Z11C16Ac and a higher amount of Z7C12Ac and Z9C12Ac compared to the corn strain females. Z9C14Ac was the major compound for both strains. Cruz-Esteban et al. (2018) found that in a Mexican FAW population, the major compound was Z9C14Ac followed by Z11C16Ac and Z7C12Ac. Tumlinson et al. (1986) also reported Z9C14Ac as the major component, but Z7C12Ac was emitted higher in proportion than Z11C16Ac in FAW females from Florida, similarly African population releases higher concentration of Z7C12Ac (Haenniger et al. 2020). Another example of regional variation in the pheromone blend is the presence of (*E*)−7-dodecenyl acetate (E7C12Ac), which has been detected exclusively in the pheromone glands of a Brazilian population (Batista-Pereira et al. 2006).

It is important to note that the variation found between the two populations could partly result from using different sampling methods, i.e., collecting volatiles using solid-phase microextraction (SPME) (Cruz-Esteban et al. 2018) and extracting pheromone compound from the pheromone glands of female moths (Tumlinson et al. 1986). Total seven pheromone compounds were reported by Tumlinson et al. (1986), which were extracted from pheromone glands and volatiles from female FAW in Florida, whereas Batista-Pereira et al. (2006) extracted eight pheromone compounds from the gland of Brazilian populations. In contrast, Cruz-Esteban et al. (2018) and Lima and McNeil (2009) found three pheromone compounds Z9C14Ac, Z7C12Ac and Z11C16Ac from virgin female FAW.

We used both headspace and gland extraction techniques to quantitively and qualitatively identify the components emitted by females during their calling and those present in their pheromone glands. Both methods confirmed Z9C14Ac as the major compound. In the headspace extract, the second most abundant compound was Z11C16AC followed by Z7C12Ac and Z9C12AC, a similar trend that was also observed in gland extraction analysis. The findings on the pheromone gland content composition align with previous studies where they reported Z9C14Ac as a major pheromone compound regardless of the sampling methods (Tumlinson et al. 1986; Lima and McNeil 2009; Cruz-Esteban et al. 2018). However, these studies reported variations in the relative proportions of minor compounds. Additionally, it should be noted that the pheromone gland components may contain compounds with little significance to sexual attraction such as precursors, antagonizing compounds and related metabolic products. Roelofs et al. (1974) suggested that the chemical signal perceived by male moths in the field may differ substantially from the proportion of compounds in gland extracts. This highlights the importance of characterizing emitted pheromone compositions to better understand the dynamics and specificity of the pheromone communication system in target species.

The observed temporal pattern of volatile release, with higher levels between the four and six hours of the scotophase (Fig. 6), aligns with previous studies that reported a nocturnal pattern of pheromone release in FAW (Savadatti et al. 2023). During scotophase a higher pheromone titre has been found in the gland of nocturnal females (Raina et al. 1986). Possibly because all reproductive activities of nocturnal females usually take place at dark phase; thus, maintaining higher pheromone titre at scotophase may facilitate those activities (Raina et al. 1991; Li et al. 2015). In a closely related species *Spodoptora litura* a higher pheromone titre in the glands of females were observed during the first scotophase, followed by a sharp decline (Lu et al. 2017). The pattern of pheromone release, common among many moth species, maximizes the chances of attracting mates by coinciding with peak male activity. According to Baker and Cardé (1979), the circadian rhythm governing the emission of pheromones plays a pivotal role in facilitating the synchronization of mating behaviours among moths. This synchronization allows individuals to respond to diurnal and seasonal fluctuations in environmental conditions, ensuring successful mate detection. Releasing pheromones during specific times of the day or night may serve an adaptive function, as it can lower the likelihood of predation by minimizing exposure to visually oriented predators (Papke et al. 2001).

We found that release of headspace volatiles was significantly higher in virgin females compared to mated females. While virgin females release higher compounds to attract males and secure a first mating, the reduction in release by mated females may act as a mechanism to avoid unnecessary attraction from additional males once mating has already occurred. Interestingly, we found that mated females retained higher levels of pheromone gland compounds in their glands compared to virgin females. Previous studies suggested that the composition of the pheromone gland and the volatile compounds released by female moths can vary considerably with female mating status (Acin et al. 2010; Lu et al. 2017). Mated females exhibited significantly higher pheromone titres in their glands compared to virgin females (Lu et al. 2017). Studies on moths revealed that sex pheromone production is governed by a pheromone biosynthesis activating neuropeptide (PBAN). During mating, male's factor transferred to the female interacts with the release mechanism of PBAN, inhibiting further production of the pheromone (Raina 1988). A specific polypeptide in the male reproductive system that, when introduced to virgin female moths, leads to the reduction of their sex pheromone levels (Kingan et al. 1995). In the present study, following mating and transmission of male-originated gland fluids, mated females decrease in pheromone gland contents release, while the amount of compounds in their glands remains high. The simplest explanation for the higher pheromone found in the glands of mated females compared to virgin females is that virgin females release higher levels of pheromones to increase their chances of attracting males and secure a first mating (Li et al. 2012; Yu et al. 2014) resulting in a sharp decrease in the pheromone content of the glands, despite pheromone production is ongoing. Therefore, we hypothesize that mating and male accessory gland fluids suppress female calling behaviours, significantly slowing the release of sex pheromones and thus resulting in higher sex pheromone titres in the pheromone glands of mated females compared with virgin females. Additionally, mated females may become more selective in subsequent matings, conserving their pheromone resources for future reproductive events. This strategy may ensure egg fertilization while preventing unnecessary energy expenditure. Furthermore, reduction in pheromone release may serve as an adaptive response to minimize fitness and physiological costs associated with pheromone production (Foster and Johnson 2012; Harari and Hadass 2013; Gao et al. 2020). It could also function as a defensive mechanism, reducing the likelihood of parasitism and predation, as excessive pheromone release could increase visibility to natural enemies (Gao et al. 2020).

Dominguez et al. (2019) reported that the amount of pheromone stored in the glands of the females and released during calling decreases with age. The highest headspace volatile release in one-day old females suggests that they may be more attractive to potential mates and have a higher mating success rate compared to older females. We observed the maximum release from one-day old females. A sharp decline was observed for three-day old females, and the lowest release was recorded in seven-day old females. A similar trend was reported by Lu et al. (2017) in a closely related species *Spodoptera litura* females, where sex pheromone titres peaked during the first scotophase after adult emergence and gradually declined afterwards. This decline is thought to be influenced by changes in the endocrine system associated with insect aging (Rafaeli 2002; Groot et al. 2015). In corn stalk borer females, pheromone release was highest within the first two days after emergence, followed by a gradual reduction in subsequent days (Babilis and Mazomenos 1992). Torres-Vila et al. (2002) noted that in noctuids, virgin females experience a decline in the receptivity, fertility, fecundity and pheromone production as they age. Generally, pheromone release rates increase for a few days after female emergence (Greenfield 1981). A study on false codling moths reported significantly higher levels of pheromone compounds in the gland of young female moths compared to old females (Levi- Zada et al. 2019). This decline is likely due to reduced glandular activity, physiological and hormonal changes over time. These findings along with our observation suggest that pheromone production is closely linked to reproductive fitness, with younger females investing more in chemical signalling to maximize mating opportunities.

This study is the first to report variations in candidate pheromone compounds in the Australian FAW population influenced by age and mating status, confirming the identified compounds and their relative proportions. The observed variation among the compounds possibly may influence male attraction and, consequently, the efficacy of pheromone-based monitoring tools. However, without validating these results through behavioral assays and field trials, it remains uncertain whether males respond accordingly to these variations. The findings of this study provide a baseline for conducting further field studies aimed at identifying an effective lure blend based on the potential variability in FAW pheromone profiles in the Australian FAW population. Such efforts could significantly enhance the management of local FAW populations in Australian cropping systems. Overall, the findings nonetheless emphasize the significance of accounting for physiological status in further pheromone lure development and testing.

## Supplementary Information

Below is the link to the electronic supplementary material.

Supplementary file1 (DOCX 166 KB)

## Data Availability

No datasets were generated or analysed during the current study.
