# Supplementary material for: Variations in Sex Pheromone of the Australian Population of Fall Armyworm: Influence of Age and Mating Status
Source: J Chem Ecol. 2025 May 17;51(3):55. doi: 10.1007/s10886-025-01607-0 (PMC12085333; doi:10.1007/s10886-025-01607-0)
Supplement: Supplementary file 1 — Supplementary file1 (DOCX 166 KB) [file 10886_2025_1607_MOESM1_ESM.docx]

Supplementary Material for the article

Contents

Supplementary Fig. S1. Mass spectra of (Z)-7-dodecenyl acetate, (Z)-9-dodecenyl acetate, (Z)-9-tetradecenyl acetate, and (Z)-11-hexadecenyl acetate analysed with electron impact (EI) and chemical ionisation (CI). MH represents protonated molecular ion.

Supplementary Table S1. Validation parameters for the internal standard (1-octanol in aqueous solution) in headspace analysis. Additional samples were analyzed to confirm specificity and calculate relative standard deviation (RSD) values.

Mass spectra of (*Z*)-7-dodecenyl acetate (molar mass: 226.35962)

MH^+^

CI

EI

Mass spectra of (*Z*)-9-dodecenyl acetate (molar mass: 226.35962)

Mass spectra of (*Z*)-9-tetradecenyl acetate (molar mass: 254.4081)

Mass spectra of (*Z*)-11-hexadecenyl acetate (molar mass: 282.4614)

**Fig.S1**

| Validation parameter | Observation/RSD |
| --- | --- |
| Specificity | No overlapping peaks observed |
| Reproducibility | 4.31% |
| Interday variation | 6.95% |
| Intraday variation | 8.67% |

**Table S1**
